# Engineering gold nanoworms with tunable longitudinal plasmon peak in the near infrared and their refractive index sensing properties

**DOI:** 10.1039/d4ra00994k

**Published:** 2024-04-19

**Authors:** Misbah ul Ain, Asma, Rizwan Ullah, Zanjbeel Fatima, Ahsan Illahi, Waqqar Ahmed

**Affiliations:** a Materials Laboratory, Department of Physics, COMSATS University Islamabad Park Road 45500 Pakistan w.ahmed@comsats.edu.pk +92 51 9049 5305; b Research in Modeling and Simulation (RIMS) Group, Department of Physics, COMSATS University Islamabad Park Road 45500 Pakistan

## Abstract

The plasmonic properties of rod-shaped Au nanoparticles make them promising for numerous applications. The synthesis recipes for Au nanorods are well established and their longitudinal plasmon peak can be tuned over a wide wavelength range. Herein, we demonstrate that the longitudinal plasmon peak of gold NWs (NWs), which are bent nanorods, can be finely tuned in the near-infra-red region. The NWs were synthesized using a one-step reaction method. We have seen that the length and aspect ratio of NWs can be tuned by simply changing the pH of the reaction medium. Under higher pH reaction conditions, NWs with relatively smaller sizes were obtained. Similar to nanorods, NWs have a well-defined longitudinal plasmon peak, which scales linearly with their aspect ratio. Finite element analysis was used to model the optical properties of Au NWs. The simulated results matched well with the experimental spectra. The synthesized NWs have shown good refractive index sensitivities (RIS). The RIS of NWs increased with an increase in their aspect ratio. A maximum sensitivity value of 542 nm per RIU, was obtained for NWs with the plasmon peak at 1033 nm. The RIS values are comparable to that of Au nanorods and bipyramids.

## Abstract 2

Gold nanoworms (NWs), synthesized in a one-step reaction with size controlled by pH, show tunable plasmon peaks in the near-infrared. Finite element analysis validates their optical properties, and they exhibit sensitive refractive index sensing.

## Graphical abstract

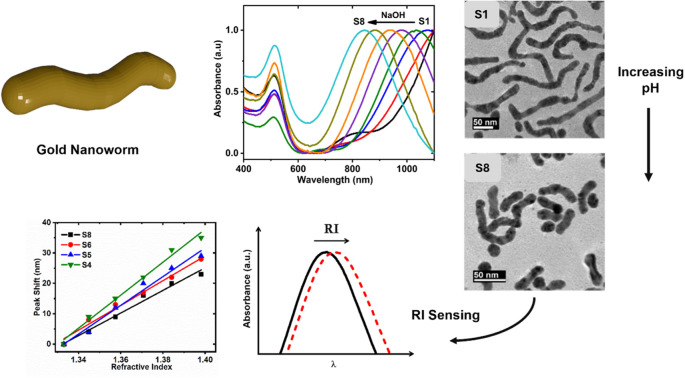

## 1. Introduction

Au nanoparticles have attracted great attention owing to their interesting optical properties that make them promising for numerous applications in catalysis, biomedicine, surface-enhanced Raman scattering (SERS) based trace detection, and refractive index sensing.^1–8^ The optical properties of Au nanoparticles originate from the localized surface plasmon resonance, which is the collective excitation of free electrons with electromagnetic (EM) waves. The surface plasmon resonance is excited when the incident EM wave is in resonance with the natural frequency of oscillation of the free electrons. The resonance condition depends sensitively on the size, shape, and surface roughness of the NPs as well as the refractive index of the medium in which they are dispersed or immobilized.

For spherical nanoparticles, there is one plasmon peak, while the anisotropic nanoparticles can have more than one plasmon peaks owing to the shape anisotropy which may result in different resonance frequencies along different axes. Among the anisotropic Au nanoparticles, the nanorod is by far the most studied shape as it has an intense longitudinal surface plasmon resonance (LSPR) peak which is tunable to a wide wavelength range in the visible and near-infrared region.^3,5,7,9^ This makes nanorods interesting for numerous applications such as cancer therapy, cell imaging, targeted drug delivery, SERS and refractive index sensing.^1,5,6,8,10^ However, tuning of the LSPR on a specific wavelength is generally required for a specific application. For instance, for biological applications, it is preferred to have the LSPR within the therapeutic window where the human body is relatively transparent to electromagnetic waves.^2,4^ Therefore, one of the key interests in the synthesis of nanorods is tuning their LSPR peak position.

Seed-mediated synthesis is the most studied method for the synthesis of anisotropic Au nanoparticles especially Au nanorods.^11,13,14^ In recent years, thousands of papers have been published detailing different aspects of gold nanorods synthesis.^5,7,11–16^ The most commonly used analytes for the synthesis are HAuCl_4_, CTAB, ascorbic acid and AgNO_3_, in addition to the seed particles. The aspect ratio of Au nanorods can be easily tuned by tuning the concentration of these reagents.^17–20^ Additionally, it has been noticed that the order in which these reagents are added to the growth solution can significantly alter the synthesis.^21–23^ We have recently shown that worm-shaped Au nanoparticles, which actually are ‘bent’ nanorods, can be synthesized by switching the addition of reagents with regards to the synthesis recipe of gold nanorods.^21^ The NWs have shown intense LSPR peak, similar to nanorods. Additionally, they showed better electromagnetic field enhancement for SERS, owing to the presence of surface roughness and bends.^24^ Moreover, the surface roughness and bends are also expected to provide improved catalytic performance due to the presence of a high concentration of low coordination atoms and better refractive index sensing owing to the better confinement of charges. Therefore, optimizing the synthesis of Au NWs has great potential. The LSPR peak of previously synthesized NWs was at 1050 nm and beyond.^21,24^ Herein, we demonstrate that the LSPR peak of Au NWs can be finely tuned from 844 nm to further in near-infrared simply by tuning the pH of the reaction conditions in a one-step synthesis. The synthesized NWs have shown linearly scalable LSPR with the aspect ratio. Moreover, the NWs have shown good refractive index sensing which depends sensitively on their LSPR peak position.

## 2. Methods

### 2.1 Materials

HAuCl_4_ (99.99%), ascorbic acid (≥99%), NaOH (≥98%), AgNO_3_ (≥99%) and CTAB (≥99%) were purchased from Sigma-Aldrich. All solutions were made with deionized (DI) water. The glassware was cleaned with *aqua regia* and thoroughly washed with DI water.

### 2.2 Synthesis of NWs

The synthesis of Au NWs was carried out by a facile one-step method. First, a CTAB solution (5 ml, 0.2 M) was prepared by adding CTAB powder to DI water. This solution was then heated on hotplate at 60 °C, under continuous stirring, to ensure complete dissolution of the CTAB powder. After the CTAB was fully dissolved, the solution was allowed to cool down to 25 °C. Following this, HAuCl_4_ (25 μl, 0.1 M) solution was added to the clear CTAB solution and stirred until a clear yellow solution was obtained. Ascorbic acid solution (30 μl, 0.1 M) was added to this yellowish solution which made the solution colorless. This was followed by the addition of different concentrations of 0.1 M NaOH solution, with the samples labelled as S1–S8 based on the volume of NaOH used. Further details of volumes of NaOH used and the resultant pH of the reaction medium have been provided in Table 1. In the final step, AgNO_3_ solution (12 μl, 0.1 M) was added quickly under continuous mixing of the solution which caused the color of the solution to change from colorless to light brown, indicating the formation of NWs. The AgNO_3_ solution was added immediately after the addition of NaOH. In case solution is left alone after the addition of NaOH, a reddish color appears within a few minutes, depending upon the pH of the reaction medium (for higher pH the color appears faster), indicating the formation of nanoparticles. In this case spherical nanoparticles are formed. Conversely, when AgNO_3_ is added immediately after the addition of NaOH, light brown color appears within seconds, which intensifies with time, and NWs are formed. The solution was left undisturbed at 25 °C overnight to allow the completion of growth.

**Table 1:** Details of the synthesis parameters and results for different samples

Sample	NAOH (μl)	pH	Length (nm)	Width (nm)	Aspect ratio	LSPR peak (nm)
S1	200	7.47	244 ± 62.2	19 ± 12.6	16.9 ± 6.4	>1100
S2	300	10.46	—	—	—	>1100
S3	330	10.65	99 ± 18.7	16 ± 1.1	6.2 ± 1.4	1075
S4	360	10.98	72 ± 11.7	13 ± 1.0	5.5 ± 1.1	1033
S5	390	10.95	65 ± 11.5	13 ± 1.2	5.1 ± 1.2	981
S6	420	11.10	57 ± 11.6	13 ± 1.0	4.5 ± 1.2	941
S7	450	11.24	—	—	—	882
S8	480	11.32	52 ± 11.8	15 ± 1.8	3.6 ± 1.1	844

### 2.3 Characterization

The UV Vis absorption spectra were performed to confirm the initial formation of desired nanoparticles using a Shimadzu UV-1280 spectrophotometer and their morphology was further studied using JEOL JEM-2100 transmission electron microscope (TEM). UV-Vis spectra of NWs were obtained without purification. For TEM analysis, samples were centrifuged at 12 000*g* twice. After the first centrifugation step, the precipitates were redispersed in DI water. After the second centrifugation step, the precipitates were redispersed in a 5 times lower volume of water to concentrate the samples. Subsequently, for the TEM analysis, 10 μl of each sample was placed on a TEM grid and was allowed to dry in ambient conditions.

### 2.4 Refractive index sensing

Water–glycerol mixtures with varying volume percentage of glycerol were prepared to ensure the change in the refractive index of the surrounding medium. The percentage of glycerol in the solution was increased from 0% to 50% at 10% intervals. The as-prepared Au NWs were first centrifuged at approximately 12 000*g* for 12 min, which caused the NWs to settle at the bottom. The supernatant was then carefully removed, and the precipitates were redispersed in the water–glycerol mixtures. The absorption spectrum of each solution was obtained and longitudinal plasmon peak shift relative to the initial longitudinal plasmon resonance wavelength was calculated. The peak shift was then plotted as a function of refractive index and sensitivity was found by linear fitting of the data points.

### 2.5 Simulations

COMSOL Multiphysics wave optics module was used to perform finite element simulations in the frequency domain using scattered field formulation. The worm shaped NPs with varying aspect ratios were modeled in a 3D spherical computational space surrounded by a perfectly matched layer (PML). Computations were performed for a single Au NW immersed in a homogenous, non-absorbing medium of dielectric function *ε*_1_. Johnson and Christy data with linear interpolation was used to model the dielectric properties of Au NWs.^25^ The computational space was discretized into a triangular finite element mesh with a size of 0.2*λ*, using a built-in algorithm in COMSOL.

A finite element method (FEM) based study for optical properties of NWs was carried out. An incident electric field ***E***_**i**_ with an amplitude 
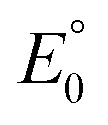
 propagating along *z*-axis and with polarization angle *θ* w.r.t *x*-axis was used to excite the NWs.1
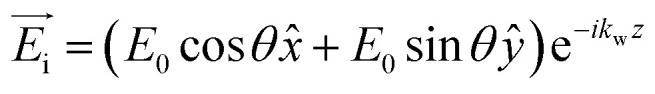
where, *k*_w_ = *nk*_0_ and *k*_0_ = *ω*/*c*. Here, *n* represents the refractive index of the medium, *k*_0_ is the wave number in vacuum, *ω* represents the angular frequency of the wave and *c* is the speed of light in vacuum. In order to compute the scattering cross section, *σ*_s_, we took a surface integral across the nanoparticle using,2
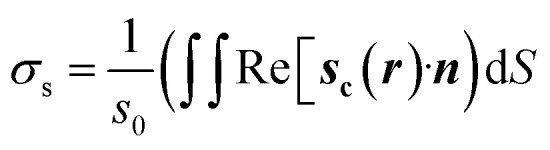
where 
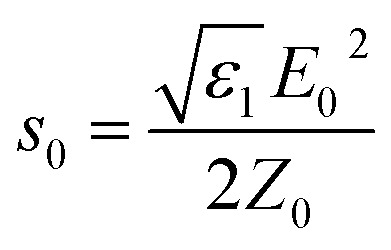
 is the power density of incident field, ***s***_**c**_ is the scattered field and *Z*_0_. = 376.73 Ω is the characteristic vacuum impedance. Similarly, the absorption cross section, *σ*_a_, was determined by taking a volume integral inside the nanostructure.3
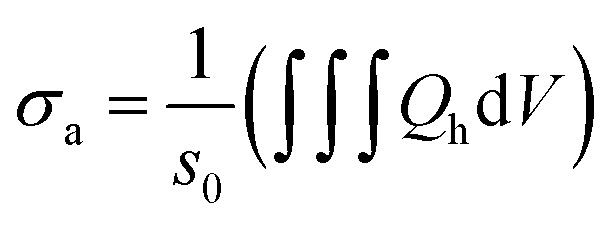
and4
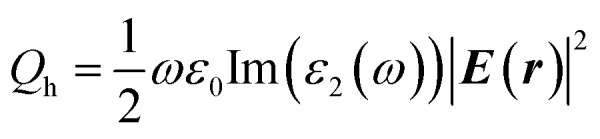
where *Q*_h_ is the total power dissipation density. *ε*_2_(*ω*) represents the relative wave dielectric function of the particle. The extinction cross-section *σ*_ext_, was then calculated as

5 *σ*_ext_ = *σ*_a_ + *σ*_s_

## 3. Results and discussion

**Fig. 1:**
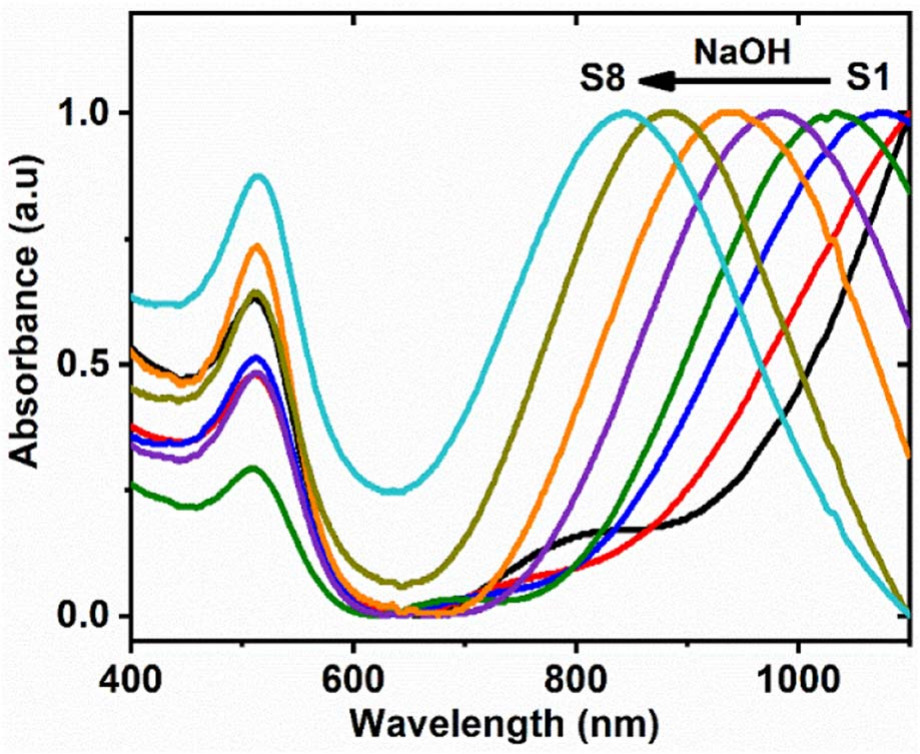
Absorption spectra of NWs synthesized at different pH (arrow indicates the direction of increasing NaOH concentration).

To study the optical properties of NWs, UV-Vis spectra were taken. Fig. 1 depicts the UV-Vis spectra of samples synthesized at different pH. All samples seem to have two well-defined distinct peaks; the longitudinal plasmon peak, and a transverse plasmon peak, similar to rod-shaped nanoparticles. With the decrease in the concentration of NaOH, a red shift in the LSPR was observed. The LSPR shifts from 844 nm to 1072 nm with decreasing NaOH concentration for samples S8 to S3, while for samples S1 and S2 it was beyond 1100 nm. The red shift in the plasmon peak indicates the formation of NWs of longer lengths and higher aspect ratios.

**Fig. 2:**
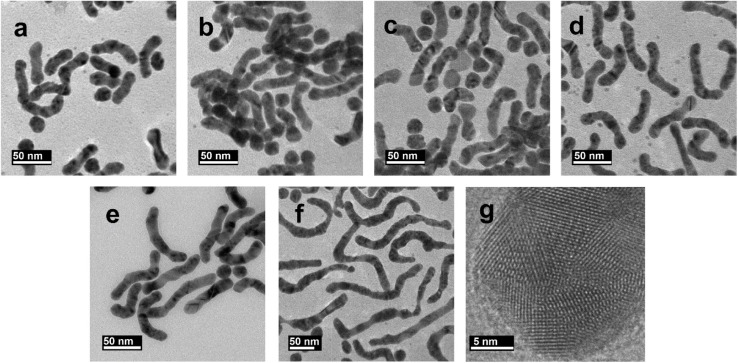
TEM images of samples (a) S8, (b) S6, (c) S5, (d) S4, (e) S3 and (f) S1; (g) high resolution TEM image of a NW showing its polycrystalline nature.

Fig. 2a–f shows the TEM images of six different samples of NWs. It is evident from the TEM images that each NW has a morphology with several bends and uneven width. Moreover, an increase in the length and aspect ratio of particles with decreasing NaOH concentration is apparent from these figures. The length, width and aspect ratio of NWs have been provided in Table 1. The measurements of length and width were conducted by selecting an average of 100 NWs from the TEM images. For the length, contour length from tip to tip was measured. As for the width, it was measured at various regions along each NW to account for potential variations in width. Then, the average width for each NW was calculated. Finally, the average length and width of 100 NWs were calculated. The values of length, width and aspect ratio for different samples are given in Table 1. Fig. 2g depicts the high-resolution TEM image of a NW. In this case, the polycrystalline morphology of NWs is clearly evident.

The polycrystalline structure and worm-shaped morphology of NWs indicate that the growth in this case is due to the coalescence of particles in the growth solution. This is clearly different from the growth of well-known Au nanorods which grow by deposition of Au atoms on seed particles *via* an atom-by-atom deposition.^11,13^ However, the reagents used for growth in both cases are very similar. The only difference is the order in which the reagents are added to the growth solution. In both cases, HAuCl_4_ is added to the CTAB solution, which initially leads to ligand exchange, resulting in the formation of AuBr_4_^−^, and subsequently CTA–AuBr_4_^−^ complex is formed. The complex formation cathodically shifts the redox potential of Au ions.^26^ For Ag-assisted growth of Au nanorods, at this stage, AgNO_3_ is added to the growth solution resulting in the formation of CTA–Ag–Br complex which serves as the key parameter in controlling the growth and subsequently, the aspect ratio of NRs.^43^ This is followed by the addition of ascorbic acid which reduces Au ions from Au(iii) to Au(i) resulting into the formation of CTA–AuBr_2_^−^.^11^ Moreover, Ag cannot be reduced from CTA–Ag–Br complex at this stage due to its lower reduction potential. As ascorbic acid is a weak reducing agent in acidic reaction conditions, it cannot completely reduce either Au(i) or Ag(i) from their respective complexes. For further reduction of Au(i) and Ag(i), the catalytic effect of seed particles is needed.^27^ However, the reduction capabilities of ascorbic acid increases with the increase in the pH of the reaction medium. At alkaline pH values, ascorbic acid has demonstrated the ability to entirely reduce Au ions, leading to the formation of well-defined Au nanoparticles.^28,44^

For the growth of NWs, AgNO_3_ is added after the addition of ascorbic acid in the solution. The addition of ascorbic acid in this case also results in the change of the color of the solution from yellowish to colorless signaling the partial reduction of Au(iii) ions to Au(i). The subsequent addition of NaOH will result in the appearance of a reddish color, which indicates complete reduction of Au ions and formation of Au nanoparticles. Notably, higher pH values result in a swifter emergence of color. For instance, for pH values of 7.47 and 11.32, the color appeared in approximately 3 min and 1 min, respectively, after the addition of NaOH. Intriguingly, the addition of AgNO_3_ immediately following NaOH accelerates the color change. Even when no NaOH was added in the solution (pH value was around 2 and solution remained colorless for days), the addition of AgNO_3_ triggered the nucleation of nanoparticles. This seems to suggest that the addition of AgNO_3_ causes nucleation. It is likely that ascorbic acid already present in the growth solution reduces the incoming free Ag(i), before it can make complex, forming Ag seed particles, as proposed in a previous report.^29^ After the formation of nuclei, the autocatalytic reduction of Au(i) on their surfaces initiates the growth process. The distinctive worm-shaped morphology and polycrystalline structure of NWs indicate their formation through the coalescence of nanoclusters, as opposed to the conventional atom-by-atom growth observed in the development of nanorods. As evident from the data presented in Table 1, the longest NWs were synthesized with a pH of 7.4. It is interesting to note that NW formation requires a basic reaction medium. The reduction in the aspect ratio with the higher pH reaction conditions has been previously reported for Au nanorods as well.^45^ Only spherical nanoparticles have been observed to form with this growth methodology.^22^

**Fig. 3:**
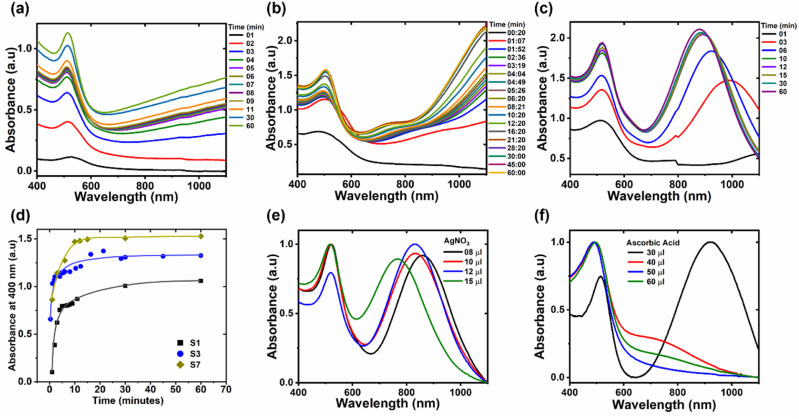
(a) UV-Vis absorption spectra of S1, (b) S2, and (c) S7 with time. (d) Change in the absorbance value at 400 nm with time for the three samples; the lines passing through the data are a guide to the eye. (e) Effect of the concentration of AgNO_3_ (0.1 M) on the synthesis of sample S8 and (f) ascorbic acid (0.1 M) on the synthesis of sample S6.

To gain a better insight into the influence of pH on growth kinetics, we employed UV-Vis spectroscopy to study the growth evaluation of samples, S1, S2 and S7, synthesized at pH values of 7.47, 10.46 and 11.25, respectively. The UV-Vis spectra of these samples were recorded at different growth stages, as illustrated in Fig. 3a–c.

Initially, a discernible peak around 500 nm appeared within the initial minute of growth for all samples, marking the start of first growth stage and indicating the formation of spherical nanoparticles. Subsequently, a longitudinal peak emerged, indicating symmetry breaking and onset of shape anisotropy. This is the second stage of growth and is likely attributed to the coalescence of nanoparticles. Notably, as the time progressed, the longitudinal peak experienced a blue shift (Fig. 3c), indicating the reduction of the aspect ratio of NWs. This is the final growth stage and is attributed to autocatalytic reduction of Au(i) on pre-existing NWs, which leads to the reduction of their aspect ratio. Similar reduction in aspect ratio with growth time has been previously reported for gold nanorods.^30^

**Fig. 4:**
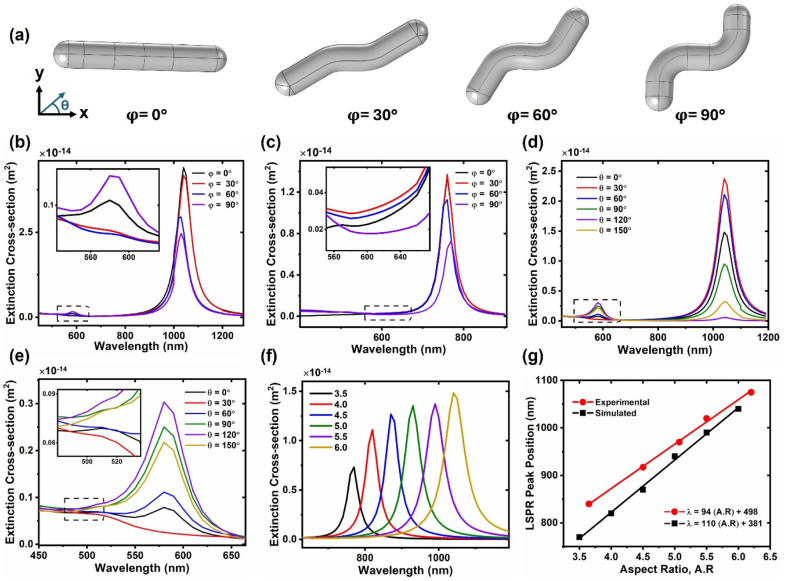
(a) NW model used for simulations; the width of the NW was fixed at 15 nm while the length was changed to change the aspect ratio, *Θ* is the polarization angle of the incident wave w.r.t to the *x*-axis, *φ* is the NW's bend angle w.r.t the central segment; (b) simulated extinction spectra of a single NW at a polarization angle of *Θ* = 0° for different bend angles for the NW of aspect ratio 6 and (c) aspect ratio 3.5. (d) Simulated extinction spectra of a single NW having *φ* = 90° at different polarizations angles; (e) magnification of dotted box region in (c) and (f) simulated LSPR of a single NW with different aspect ratios for *Θ* = 0°, *φ* = 90°; legend represents the values of aspect ratios; (g) comparison of experimental and simulated LSPR position w.r.t the aspect ratio (A.R); the equations representing the linear fitting in both cases have been provided in the legend.

Furthermore, monitoring the absorbance change at 400 nm provided insights into the presence of Au(0) in the solution, offering valuable information on Au(i) reduction and particle growth dynamics.^26,31^Fig. 4d illustrates the absorbance change at 400 nm for the three samples. Remarkably, the absorbance plateaued within 10 minutes for sample S8 and slightly later for sample S2. However, for sample S1, significant growth persisted even when one hour of growth time elapsed. This verifies that the samples synthesized at higher pH values have accelerated growth rate.

Other variables that could influence the growth kinetics are the concentrations of ascorbic acid and AgNO_3_ in the growth solution. The values of these reagents provided by us in Section 2.2 are the optimized values. We have observed that the NW synthesis is very delicate and deviation from these values leads to degradation of the growth yield. Fig. 3e and f illustrates the impact of varying the concentration of AgNO_3_ in the synthesis of sample S8, and ascorbic acid in the synthesis of sample S6. From these spectra, we deduce that the optimal volumes for synthesis are 30 μl (0.1 M) of AgNO_3_ and 12 μl (0.1 M) of ascorbic acid, as detailed in Section 2.2.

In order to model the optical properties of Au NWs, COMSOL simulations based on finite element analysis were carried out. Fig. 4a represents the morphology of NW used for simulations. The NW consists of three segments of approximately equal length. The central segment is oriented parallel to the *x*-axis. The width of NW was fixed at 15 nm in each case, while the contour length was changed to change the aspect ratio. Fig. 4b depicts the extinction spectra of a single NW with an aspect ratio of 6 and length of 90 nm, at different bend angles (*φ*), with respect to the central segment, using an EM wave polarized along the *x*-axis. There is a clear decrease in the LSPR peak intensity as the bend angle of the NW increases from 0° to 90°. However, interestingly, the peak position does not change much with the bend angle. This seems to suggest that the variation in the bend angles of NWs within the NW suspension does not have a huge influence on the LSPR peak width. An additional peak can be observed at a wavelength of 580 nm, as depicted in Fig. 4b. This peak is absent for relatively smaller NWs (aspect ratio of 3.5 and length of 52.5 nm), as depicted in Fig. 4c. This seems to suggest that this peak arises due to higher order quadrupole mode. Fig. 4d shows the extinction spectra of a single NW (aspect ratio of 6 and length of 90 nm) with the bend angle of 90° simulated at different polarization angles of the incident electromagnetic wave. As the polarization direction of the incident electromagnetic wave changes from the longitudinal to the transverse direction, the LSPR peak intensity clearly decreases.

Fig. 4f depicts the optical spectra of NWs with different aspect ratios using an EM wave polarized along the *x*-axis. The length of NWs varies from 52.5 nm to 90 nm, while the width was kept constant at 15 nm. Expectedly, there is a clear increase in the extinction cross-section with the increase in aspect ratio. Moreover, the LSPR peak redshifts significantly with the increase in aspect ratio. Fig. 4g depicts the comparison of the variation of the LSPR peak position with the aspect ratio of the simulated spectra and the experimental spectra. As evident from the figure, linear trends of aspect ratio *vs.* LSPR peak position have been observed. In both cases the values of regression coefficient are greater than 0.99, which verifies an excellent linear fit. Therefore, the aspect ratio of Au NWs can be estimated with confidence simply with the information of their LSPR peak position. Moreover, the simulated and experimental LSPR peak positions have a good match. However, the simulated peaks appeared at slightly lower wavelengths than the experimental peaks. This difference may be attributed to the fact that simulations were performed for a single NW of a certain length and width. On the other hand, experimental samples contain NWs of varying lengths, so an average length was considered. The contribution of longer NWs to the extinction spectrum is greater, due to their larger extinction cross-section, compared to their smaller counterparts. This results in an LSPR peak at a slightly longer wavelength than what is obtained for a NW with an average length or aspect ratio. Nonetheless, the model presented here provides optical properties that are in good agreement with the experimental spectra. The linear trend obtained in this work is very similar to the ones calculated for Au nanorods using Gans approximation (*λ* = 94.5 A.R + 396.5)^32^ and discrete dipole approximation (*λ* = 98 A.R + 396.5).^33^

### 3.1 Refractive index sensing

Plasmonic nanoparticles are well known for their excellent refractive index sensing. The LSPR peak is sensitive to the permittivities of the surrounding medium. Generally, the refractive index sensitivity (RIS) depends on the LSPR peak position or the aspect ratio of nanoparticles.^34^ Particles having longer LSPR peak positions show better sensitivities. Moreover, in some reports, particles with sharp tips *e.g.*, nanorods, nanostars, and nanobipyramids have been reported to show better RIS owing to the charge confinement.^35–40^

**Fig. 5:**
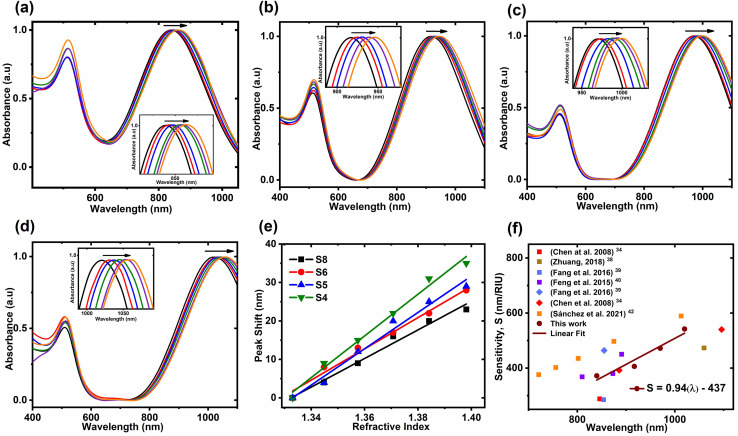
UV-Vis spectra of samples (a) S4, (b) S5, (c) S6 and (d) S8 immersed in water–glycerol solutions (inset shows the zoomed LSPR peaks); (e) the linear fit of LSPR peak *vs.* refractive index of each sample. (f) Comparison of RIS of NWs (circles), nanorods (squares) and bipyramids (diamonds); the square symbol represents the data for nanorods, and the diamond symbol is for NWs, and the linear fitting is for the data of Au NWs.

To test the RIS of the NWs, they were dispersed in mixtures of water (refractive index = 1.3334) and glycerol (refractive index = 1.4746) solutions, with varying refractive indices. Fig. 5a–d depicts the UV-Vis spectra of NWs in water–glycerol solutions. The arrow indicates the direction of increasing RI. There is a prominent shift in the LSPR peak position with the increase in the refractive index in all cases. This shift seems to be more pronounced for samples with higher aspect ratio or LSPR peak position at longer wavelengths. The slope of the linear fit corresponds to the sensitivity values of 542, 472, 406 and 371 nm change in LSPR peak position per unit change in refractive index (RIU) for samples S4, S5, S6, and S8, respectively. The peak shift is plotted against the refractive index of the medium in Fig. 5f, which demonstrates a good linear relationship for each sample. Moreover, Fig. 5f compares our RI sensing results with that of other one-dimensional gold nanoparticles, *i.e.*, nanorods and bipyramids. The data verifies that NWs possess similar, if not higher, sensitivities compared to nanorods and bipyramids of similar LSPR peak positions. Moreover, there is a linear correlation between the LSPR peak position of Au NWs and their refractive index sensing capability. Similar linear relationships have been reported previously for other particle shapes.^41^

While our NWs exhibit sensitivities comparable to nanorods and bipyramids with similar LSPR peak positions, it is crucial to consider the figure of merit (FOM) in refractive index studies. The FOM, defined as the ratio of sensitivity to the full width at half maximum (FWHM), is an important factor in assessing the performance of plasmonic sensors. In our study, we observed that the FOM decreases with the aspect ratio of the NWs with FOM values of 1.86, 1.70, 1.50 and 1.8 for samples S4, S5, S6 and S8, respectively. These FOM values are higher than previously reported FOM values for nanostars.^34,36^ However, when compared to nanorods and bipyramids of similar aspect ratios, the FOM values of NWs are inferior.^34,42^

## 4. Conclusion

In summary, we have demonstrated that a one-step facile recipe can be used for tuning the aspect ratio of worm-shaped Au nanoparticles. The aspect ratio can be changed by simply changing the NaOH concentration in the reaction medium. At higher pH, NWs with a relatively smaller aspect ratios were obtained. The change in the aspect ratio resulted in change of the LSPR peak position, which can be tuned over a wide wavelength range in the infrared. The LSPR peak position scales linearly with the aspect ratio of NWs, similar to nanorods. By using finite element analysis, we were able to model the optical properties of Au NWs. The simulated spectra agreed well to the experimental spectra. Furthermore, NWs showed good refractive index sensitivity which increased with their aspect ratio. A maximum sensitivity of 542 nm per RIU was obtained for NWs with the LSPR peak at 1033 nm. When compared with other one-dimensional Au nanoparticles with similar LSPR peak positions, Au NWs showed comparable refractive index sensitivities. The results presented here would enable researchers to utilize NWs for specific applications.

## Conflicts of interest

There are no conflicts to declare.

## Supplementary Material
